# Does a second course of low-frequency repetitive transcranial magnetic stimulation enhance neuroplasticity and motor function in children with hemiplegic cerebral palsy? Results of a prospective study

**DOI:** 10.3389/fnhum.2026.1829142

**Published:** 2026-04-29

**Authors:** Fani Galabova-Petrova, Ivan Ivanov

**Affiliations:** 1 Department of Pediatrics, UMHAT St. George, Plovdiv, Bulgaria; 2 Department of Pediatrics, Faculty of Medicine, Medical University of Plovdiv, Plovdiv, Bulgaria

**Keywords:** cerebral palsy, cortical excitability, hemiplegia, interhemispheric inhibition, motor function, neuroplasticity, pediatric rehabilitation, repetitive transcranial magnetic stimulation

## Abstract

**Background:**

Repetitive transcranial magnetic stimulation (rTMS) has emerged as a promising neuromodulation technique for enhancing motor recovery in children with hemiplegic cerebral palsy. However, long-term effects of repeated rTMS courses remain unclear, as existing studies have focused primarily on short-term outcomes following a single treatment course, with limited data on whether benefits decline, persist, or can be restored by a second course.

**Objective:**

To investigate the immediate and sustained neurophysiological and functional effects of two courses of low-frequency rTMS applied to the contralesional motor cortex in children with hemiplegic CP.

**Methods:**

Fifteen children (mean age 9.5 ± 2.8 years; GMFCS I-II, MACS I-II) received two courses of 1 Hz rTMS separated by 3 months. Each course consisted of 10 daily sessions at 90% resting motor threshold, targeting the contralesional motor cortex. Assessments at six time points included neurophysiological parameters (motor threshold MT; motor evoked potentials (MEP) latency and amplitude; cortical silent period, CSP) and functional tests (Finger Tapping, FT; Box and Blocks, BB; Melbourne Assessment, MA; Timed Up and Go, TUG; 1-Minute Walk Test, Berg Balance Scale, BBS).

**Results:**

Following the first rTMS course, MT decreased by 32% (upper limb) and 29% (lower limb); MEP latency shortened by 12 and 9%, respectively; MEP amplitude increased by 17 and 18%; and CSP decreased by 31 and 34% (all *p* < 0.001). Functional improvements consisted of 12% increase in FT and 8.5% in BB, 16% faster TUG, 16% greater 1-min walking distance, and 6.5% improved balance in BBS (all p < 0.001). During 3-month follow-up, effects gradually declined but remained significantly better than baseline. The second rTMS course produced similar magnitude improvements, restoring parameters to first-course post-treatment levels. No significant difference was found between neurophysiological and functional parameters 1 month after the first and 1 month after the second course, with the exception of progressive prolongation CSP.

**Conclusion:**

Repeated courses of low-frequency rTMS were associated with reproducible within-subject improvement in neurophysiological and functional measures in children with hemiplegic cerebral palsy. These effects appeared to partially decline over 3 months, and a second course was associated with restoration of treatment benefits without generating further major cumulative gains.

## Introduction

Hemiplegic cerebral palsy (CP) represents one of the most common subtypes of CP, characterized by unilateral motor impairment resulting from early brain injury during critical periods of neural development (Rosenbaum et al., 2007; Graham et al., 2016). The motor deficits in hemiplegic CP arise not only from the structural lesion itself but also from maladaptive neuroplastic changes in the developing brain, particularly abnormal interhemispheric interactions between left and right motor cortices (Eyre et al., 2007; Staudt, 2010). Following early unilateral brain injury, reorganization of the contralesional (non-lesional) hemisphere often results in altered patterns of interhemispheric inhibition, with the contralesional motor cortex exerting excessive transcallosal inhibition onto the ipsilesional (lesional) hemisphere (Kirton and deVeber, 2006; Murase et al., 2004). This imbalance in interhemispheric inhibition has emerged as a key target for therapeutic interventions aimed at rebalancing cortical activity and facilitating functional motor recovery (Murase et al., 2004; Takeuchi and Izumi, 2012; Hummel and Cohen, 2006).

Transcranial magnetic stimulation (TMS) provides objective neurophysiological markers that can quantify changes in corticospinal excitability and intracortical circuits. Key parameters include resting motor threshold (RMT), which reflects membrane excitability of corticospinal neurons; motor evoked potential (MEP) amplitude and latency, which indicate the strength and speed of corticospinal transmission; and cortical silent period (CSP), which reflects GABAergic intracortical inhibition (Rossini et al., 2015; Ziemann et al., 2015; Zewdie and Kirton, 2016). These measures offer mechanistic insights into how rTMS modulates cortical function and may predict or correlate with functional motor improvements (Di Lazzaro and Rothwell, 2014; Stinear et al., 2017).

Repetitive transcranial magnetic stimulation (rTMS) is a non-invasive brain stimulation technique that modulates cortical excitability through the application of repetitive magnetic pulses over specific cortical regions (Hallett, 2007; Pascual-Leone et al., 1998). Low-frequency rTMS (typically ≤1 Hz) produces inhibitory effects on the stimulated cortex, while high-frequency protocols (≥5 Hz) have facilitatory effects generally (Fitzgerald et al., 2006). In the context of hemiplegic CP, low-frequency rTMS applied to the contralesional motor cortex aims to reduce its hyperexcitability, thereby decreasing transcallosal inhibition and allowing greater activation of the ipsilesional hemisphere and improved motor control of the paretic limb (Kirton et al., 2008; Gillick et al., 2015). Several studies in adults with stroke have demonstrated the efficacy of this approach in enhancing motor recovery (Fregni and Pascual-Leone, 2007; Nowak et al., 2009). Emerging evidence suggests similar benefits in pediatric populations with hemiplegic CP (Kirton et al., 2016; Kirton et al., 2010; Gillick et al., 2014).

Despite promising initial results, several important questions remain unsolved regarding the optimal application of rTMS in children with hemiplegic CP. Firstly, the sustainability of rTMS effects beyond the immediate post-treatment period is not well characterized, with most studies focusing on outcomes immediately after treatment or at short follow-up intervals, usually <1 month (Zewdie et al., 2020; Rajapakse and Kirton, 2013). Secondly, the supposed benefits of repeated rTMS courses separated by extended intervals remain largely unexplored in pediatric populations, despite theoretical considerations suggesting that multiple treatment episodes might consolidate and enhance neuroplastic changes (Vidal and Chen, 2020; Chen et al., 2002). Thirdly, comprehensive assessment incorporating both neurophysiological measures of cortical excitability and clinically relevant functional outcomes may provide essential data for understanding mechanisms underlying treatment effects and their translation to real-world motor abilities (Pascual-Leone et al., 2005; Stinear et al., 2012).

The present study was designed to address these gaps by conducting a pilot prospective investigation of the effects of two courses of low-frequency rTMS applied to the contralesional motor cortex in children with hemiplegic CP. We hypothesize that: (1) rTMS may be associated with measurable changes in cortical excitability parameters (decreased MT, shortened MEP latency, increased MEP amplitude, and prolonged CSP duration) reflecting the expected reduction of the hyperexcitability of the contralesional hemisphere, thus providing enhancement in the function of the ipsilesional corticospinal pathway; (2) these neurophysiological changes may induce functional motor improvements across multiple domains including hand dexterity, gross motor function, gait, and balance; (3) treatment effects may be maintained, at least partially, during follow-up periods; and (4) a second course of rTMS may, at least, restore neurophysiological and functional gains following partial decay of first-course effects, or even augment them to a higher level. By examining outcomes at six time points over an extended period of 6 months, this study provides comprehensive data on both the immediate and enduring effects of repeated rTMS interventions in pediatric hemiplegic CP.

## Materials and methods

### Participants

This prospective study included 15 children (8 males [53.3%] and 7 females [46.7%]) with mean age 9.5 ± 2.8 years (range 6–14 years) diagnosed with hemiplegic CP. Right-sided hemiparesis was present in 8 children (53.3%) and left-sided - in 7 (46.7%). All participants were recruited from the Department of Pediatrics, St. George University Hospital, Medical University of Plovdiv, Bulgaria, between January 2024 and June 2025.

All rTMS sessions and assessments were conducted at the outpatient clinic of the Department of Pediatrics, St. George e Gross Motor Function Classification System (GMFCS) level I-II (Palisano et al., 1997); (4) Manual Ability Classification System (MACS) level I - II (Eliasson et al., 2006); (5) ability to cooperate with TMS procedures and functional assessments; (6) no seizures in the previous 6 months (Zewdie et al., 2020; Rachid, 2018); (Hallett, 2007) no medications affecting cortical excitability (e.g., anticonvulsants) or stable medication regimen for at least 3 months prior to enrollment.

Exclusion criteria were: (1) contraindications to TMS (metallic implants, cardiac pacemaker) (Rossini et al., 2015; Groppa et al., 2012); (2) severe cognitive impairment preventing task comprehension (Zewdie et al., 2020); (3) uncontrolled epilepsy (Zewdie et al., 2020; Groppa et al., 2012); (4) botulinum toxin injection within 6 months prior to study entry (Kirton et al., 2016; Gillick et al., 2014); (5) orthopedic surgery within 12 months before rTMS (Kirton et al., 2016; Gillick et al., 2014).

The functional classification of participants according to GMFCS showed 12 children with level I (80%) and 3 children with level II (20%). MACS classification revealed an identical distribution: 12 children with level I (80%) and 3 with level II (20%), but with discrepancy between GMFCS and MACS levels at individual level in 2 patients (13.3%).

All 15 children completed both courses of rTMS with all time points assessments with no dropouts.

All participants continued their routine physiotherapy programs (typically 2–3 sessions per week) throughout the study period. Participants were instructed to maintain stable therapy intensity and not to initiate new interventions during the study period.

The study protocol was approved by the Ethics Committee of Medical University of Plovdiv and was conducted in accordance with the Declaration of Helsinki. Written informed consent was obtained from parents or legal guardians of all participants, and age-appropriate assent was obtained from children capable of understanding the study procedures.

### Study design

This prospective single-arm substudy examined whether a second rTMS course could restore or enhance neurophysiological and functional improvements following partial decay of first-course effects. Participants served as their own controls across six assessment timepoints.

This study represents a substudy of a larger prospective investigation (*n =* 68) examining single-course low-frequency rTMS in children with hemiplegic CP. The 15 participants were selected based on good therapeutic response to the first rTMS course (defined as ≥20% improvement in at least one neurophysiological parameter) and clinical indication for a second treatment course. Sample size was determined by clinical feasibility and therapeutic indication rather than formal *a priori* power calculation, as this was an exploratory investigation of repeated-course effects. *Post-hoc* analysis confirmed that the observed large effect sizes ( $\eta_{p}^{2}$ = 0.47–0.85) provided >80% statistical power for detecting the primary treatment effects (Cohen, 1988).

Primary comparisons: (1) Reproducibility—immediate post-treatment effects of first vs. second course (T1 vs. T4); (2) Cumulative benefit—maintenance effects at equivalent timepoints (T2 vs. T5). Primary outcome domains were motor threshold of the upper limb (representing neurophysiological response) and Box and Blocks Test (representing functional response). Secondary outcomes included MEP latency and amplitude, cortical silent period (both limbs), and additional functional measures (Finger Tapping, Melbourne Assessment, TUG, 1-Minute Walk, Berg Balance Scale).

This study was designed as a prospective, single-arm exploratory investigation without a control or sham condition; therefore, causal inference regarding treatment effects is limited.

### rTMS protocol

Repetitive TMS was delivered using a Magstim Rapid^2^ stimulator (Magstim Company Ltd., Whitland, UK) with a figure-of-eight coil (external diameter 70 mm).

The M1 representation of the first dorsal interosseous (FDI) muscle in the contralesional hemisphere was identified as the optimal stimulation site by systematic search for the motor hotspot. EMG recordings were obtained using surface Ag-AgCl electrodes (10 mm diameter) placed in a belly-tendon montage over the FDI muscle of the paretic hand, with the active electrode positioned over the muscle belly and the reference electrode over the metacarpophalangeal joint of the index finger. The ground electrode was placed on the dorsal wrist. EMG signals were amplified (×1,000), band-pass filtered (20–2000 Hz), and displayed in real-time. The hotspot was defined as the scalp position producing the largest and most consistent MEPs at the lowest stimulus intensity (Groppa et al., 2012).

Resting motor threshold (RMT) was determined according to international guidelines being the minimum stimulus intensity required to elicit MEPs in the target muscle at rest with amplitude ≥50 μV in at least 5 out of 10 consecutive trials (Groppa et al., 2012). RMT was expressed as a percentage of maximum stimulator output (Rossini et al., 2015; Groppa et al., 2012).

The rTMS protocol consisted of low-frequency stimulation at 1 Hz frequency with 90% of individual RMT intensity for 20 min per session (1,200 pulses per session), based on established protocols for contralesional motor cortex inhibition in pediatric stroke and hemiplegic CP (Kirton et al., 2008; Gillick et al., 2014). Following standardized TMS methodology, the coil was positioned tangentially to the scalp with the handle pointing posterolaterally at approximately 45° from the midline, inducing a posterior-to-anterior current flow in the underlying cortex (Rossini et al., 2015; Groppa et al., 2012).

During stimulation sessions, participants were seated comfortably with both hands resting on armrests in a relaxed, fully supinated position. To maintain engagement and minimize movement, children were permitted to watch age-appropriate videos or listen to music through headphones throughout the 20-min stimulation period. Visual monitoring confirmed continuous muscle relaxation via real-time EMG display.

Each session lasted approximately 35–40 min, including 5–10 min for setup (hotspot verification, RMT confirmation, positioning), 20 min of active stimulation, and 5 min for safety monitoring and session completion.

Each treatment course comprised 10 consecutive daily sessions, excluding weekends. Each participant received two complete courses separated by a 3-month interval, allowing sufficient time for neuroplastic consolidation and permitting assessment of both treatment effect durability and the capacity of repeated stimulation to restore the benefits of the first course (Rajapakse and Kirton, 2013; Thickbroom, 2007).

Safety protocols followed pediatric TMS guidelines (Gillick et al., 2015). Prior to each session, all metallic objects (jewelry, hairpins, glasses with metal frames) were removed. Participants wore protective earplugs or noise-canceling headphones to attenuate the coil click sound. Participants were continuously observed for adverse events including seizures, syncope, headache, neck pain, or excessive discomfort. Heart rate and oxygen saturation were monitored throughout each session.

### Neurophysiological assessment

Comprehensive neurophysiological assessments were performed at six time points: baseline prior to first rTMS course (T0), immediately after completion of the first course (T1), 1 month after the end of the first course (T2), 3 months after the first course (immediately before second course, T3), immediately after the completion of the second course (T4), and 1 month after the second course (T5). All assessments were conducted by the same experienced neurophysiologist using standardized, objective measurement protocols to ensure consistency and minimize variability.

Baseline assessments (T0) were performed 1–3 days prior to initiation of the first rTMS course.

Single-pulse TMS was used to assess corticospinal excitability parameters. Electromyographic (EMG) recordings were obtained from the first dorsal interosseous muscle of the paretic hand and the tibialis anterior muscle of the paretic leg using surface electrodes (Ag-AgCl, 10 mm diameter) in a belly-tendon montage. EMG signals were amplified (×1,000), band-pass filtered (20–2000 Hz), digitized at 5 kHz, and stored for offline analysis.

Motor threshold (MT) was determined for both upper and lower limbs as described above. For MEP assessment, 10 stimuli were delivered at 120% RMT with intertrial intervals of 5–7 s. Peak-to-peak MEP amplitude (μV) and MEP onset latency (ms, measured from stimulus artifact to MEP onset) were measured for each trial and averaged.

Cortical silent period (CSP) was measured during sustained voluntary contraction at approximately 20% of the maximum voluntary contraction. Ten stimuli at 120% RMT were delivered, and CSP duration (ms) was measured from the MEP onset to the return of continuous EMG activity, then averaged across trials.

All neurophysiological measurements were obtained from the ipsilesional (lesional) hemisphere, reflecting the corticospinal output to the paretic limbs. This approach enabled direct assessment of the effects of contralesional rTMS on the excitability and the function of the compromised cortex and motor pathway.

All neurophysiological measurements were obtained from the ipsilesional (lesioned) hemisphere, reflecting corticospinal output to the paretic limbs. This approach assumes typical contralateral corticospinal organization. While formal assessment of ipsilateral motor projections (e.g., via diffusion tensor imaging or ipsilateral MEP mapping) was not performed, the consistent therapeutic response across all participants suggests that our cohort did not include children with substantial atypical ipsilateral motor organization that would be adversely affected by contralesional inhibition (Eyre et al., 2007; Staudt, 2010).

While rTMS was delivered to the FDI hotspot, neurophysiological assessments were obtained from both upper limb (FDI) and lower limb (tibialis anterior) muscles to examine whether focal motor cortex stimulation produces spatially distributed effects across the ipsilesional motor representation, consistent with the network modulation hypothesis of rTMS effects (Fitzgerald et al., 2006).

### Functional assessment

Standardized functional assessments were administered at all six time points by a trained pediatric neurologist following the study protocol. All tests were performed in the same order at each session to minimize variability.

Finger Tapping Test: Participants performed rapid index finger tapping for 10 s with the paretic hand while maintaining wrist stability. The number of complete taps was recorded. This test measures motor speed and coordination (Reitan and Wolfson, 1993).

Box and Blocks Test (BBT): This widely used test of gross manual dexterity requires participants to grasp 1-inch wooden blocks from one compartment and transport them over a partition into another compartment as quickly as possible in 60 s using only the paretic hand. The number of successfully transferred blocks was recorded (Mathiowetz et al., 1985).

Melbourne Assessment 2 (MA2): This criterion-referenced measure assesses quality of upper limb movement in children with neurological impairments. The MA2 evaluates four dimensions: range of movement, target accuracy, dexterity (grasp., manipulation, release), and fluency (smoothness and speed). Scores range from 0 to 100% for each subscale, with higher scores indicating better performance (Randall et al., 2001). Due to the ceiling effect observed in children with MACS level I, results are reported separately for MACS I and MACS II participants.

Timed Up and Go Test (TUG): Participants stood up from a standard chair, walked 3 meters at a comfortable pace, turned around, walked back, and sat down. The time needed to complete the task was recorded in seconds. TUG assesses functional mobility, balance, and gait (Podsiadlo and Richardson, 1991).

1-Minute Walk Test (1MWT): Participants walked at their maximal comfortable self-selected speed for 1 min along a 25-meter corridor, and the distance covered was recorded in meters. This test measures functional walking capacity and endurance (McDowell et al., 2009).

Berg Balance Scale: This 14-item scale evaluates static and dynamic balance abilities across various functional tasks including sitting, standing, transfers, reaching, turning, and single-leg stance. Each item is scored 0–4 points, yielding a total score between 0 and 56, with higher scores indicating better balance (Berg et al., 1989).

The 1-Minute Walk Test and other lower limb functional measures were included to assess whether rTMS-induced modulation of interhemispheric inhibition produces functional benefits beyond the directly stimulated upper limb representation, potentially through network-level effects on overall motor cortex excitability and balance between hemispheres (Fitzgerald et al., 2006). This exploratory aim is acknowledged as a limitation given the focal FDI-targeted stimulation and absence of task-specific motor training.

Due to the single-arm design, outcome assessors were not blinded to treatment timing. However, all assessments followed standardized protocols administered in identical order at each timepoint to minimize bias.

### Statistical analysis

Descriptive statistics are presented as mean ± standard deviation (SD) for continuous variables and frequencies (percentages) for categorical variables. The Shapiro–Wilk test was used to assess normality of data distribution.

For neurophysiological and functional outcomes, repeated measures analysis of variance (ANOVA) was performed to examine the main effect of time point (T0–T5) on each outcome measure.

Repeated measures ANOVA was selected based on: (1) complete data at all six timepoints for all 15 participants (no missing data), eliminating the primary advantage of mixed-effects models; (2) sphericity assumptions were evaluated using Mauchly’s test and Greenhouse–Geisser corrections applied where violated (Field, 2018); (3) the focus on fixed time-point comparisons rather than individual growth trajectories. Nevertheless, we acknowledge that linear mixed-effects models may offer advantages for future larger studies with missing data or interest in individual-level variation.

When significant main effects were detected, *Post-hoc* pairwise comparisons with Bonferroni correction were conducted. Particular attention was paid to comparisons of: (1) baseline vs. immediate post-treatment (T0 vs. T1, T3 vs. T4); (2) immediate post-treatment vs. follow-up periods (T1 vs. T2, T1 vs. T3, T4 vs. T5); (3) decay of effects during follow-up (T2 vs. T3); and (4) effects of first vs. second course (T1 vs. T4 and T2 vs. T5).

For MA2 subscales, data are presented as median (interquartile range) stratified by MACS level due to non-normal distribution and ceiling effects in the MACS I group. Non-parametric Friedman tests were used to evaluate changes over time within each MACS subgroup.

Effect sizes were calculated using Cohen’s d for pairwise comparisons and partial eta squared ( $\eta_{p}^{2}$ ) for ANOVA main effects. Effect sizes were interpreted according to Cohen’s guidelines (Cohen, 1988). Values of $\eta_{p}^{2}$ = 0.01, 0.06, and 0.14 were interpreted as small, medium, and large effects, respectively.

Pearson correlation coefficients were calculated to explore relationships between changes in neurophysiological parameters and functional outcomes. Statistical significance was set at *p* < 0.05. All analyses were performed using SPSS version 27.0 (IBM Corp., Armonk, NY, USA), with power calculations performed in GPower 3.1 and visualizations created in R version 4.1.2.

## Results

### Adverse effects

No adverse events related to rTMS were reported during the study.

### Neurophysiological outcomes

Comprehensive neurophysiological assessments revealed significant modulation of cortical excitability parameters following both courses of rTMS (Tables 1, 2).

**Table 1:** Neurophysiological parameters across six assessment time points.

Parameter	T0	T1	T2	T3	T4	T5	F	*p*	$\eta_{p}^{2}$
Days from onset	0	30	60	120	150	180	–	–	–
MT– Upper Limb (% MSO)	52.0 ± 8.8	35.0 ± 6.0	39.7 ± 6.1	46.0 ± 7.4	34.3 ± 5.6	39.0 ± 6.3	15.10	<0.001	0.473
MT – Lower Limb (% MSO)	73.7 ± 10.1	54.7 ± 8.5	58.0 ± 8.2	66.0 ± 9.5	53.7 ± 9.2	56.7 ± 9.2	10.98	<0.001	0.395
MEP Latency – Upper Limb (ms)	22.7 ± 0.8	20.0 ± 0.9	20.9 ± 0.8	21.9 ± 0.9	20.2 ± 0.9	20.7 ± 0.9	20.08	<0.001	0.545
MEP Latency – Lower Limb (ms)	34.1 ± 0.8	30.9 ± 0.8	31.9 ± 0.8	33.4 ± 0.8	31.6 ± 0.7	32.3 ± 0.6	38.29	<0.001	0.695
MEP Amplitude – Upper Limb (μV)	268.3 ± 5.6	312.7 ± 9.6	306.7 ± 5.6	281.3 ± 8.3	305.3 ± 6.4	301.0 ± 2.8	97.99	<0.001	0.854
MEP Amplitude – Lower Limb (μV)	374.3 ± 8.8	436.7 ± 15.4	420.7 ± 14.9	389.7 ± 10.1	427.3 ± 17.9	412.0 ± 16.1	31.98	<0.001	0.656
CSP – Upper Limb (ms)	104.0 ± 35.8	83.3 ± 44.7	78.3 ± 22.8	89.0 ± 23.0	76.7 ± 23.0	87.0 ± 23.9	1.61	0.166	0.088
CSP – Lower Limb (ms)	126.7 ± 37.4	96.0 ± 35.8	100.3 ± 34.2	116.0 ± 35.0	97.7 ± 35.6	112.0 ± 35.5	1.75	0.132	0.094

Values represented by mean ± standard deviation. MT, motor threshold; MSO, maximum stimulator output; MEP, motor evoked potential; CSP, cortical silent period. T0, baseline before first rTMS course; T1, immediately post-first course; T2, 1-month follow-up after first course; T3, 3-months post-first course/immediately before second course; T4, immediately post-second course; T5, 1-month follow-up after second course (*n =* 15). F, *p* and $\eta_{p}^{2}$ describe the main effect of time investigated by repeated measures ANOVA.

**Table 2:** *Post-hoc* pairwise comparisons of neurophysiological parameters between key timepoints – *p*-value and % change.

Parameter	T0 → T1 (Course 1 effect)	T0 → T3 (Maintenance)	T2 → T3 (Decay)	T3 → T4 (Course 2 effect)	T1 vs T4 (Reproducibility)	T2 vs T5 (Enhancement)
	*p*-value % change	*p*-value % change	*p*-value % change	*p*-value % change	*p*-value % change	*p*-value % change
MT – Upper Limb	<0.001 −32.7%	0.001 −11.5%	<0.001 +16.0%	<0.001 −25.4%	1.000 −1.9%	0.986 −1.7%
MT – Lower Limb	<0.001 −25.8%	<0.001 −10.4%	<0.001 +13.8%	<0.001 −18.7%	1.000 −1.8%	0.243 −2.3%
MEP Latency – Upper Limb	<0.001 −11.6%	<0.001 −3.2%	<0.001 +4.9%	<0.001 −7.9%	1.000 +0.8%	0.324 −1.0%
MEP Latency – Lower Limb	<0.001 −9.3%	<0.001 −2.1%	<0.001 +4.7%	<0.001 −5.3%	0.010* +2.3%	0.063 +1.5%
MEP Amplitude – Upper Limb	<0.001 +16.5%	0.001 +4.8%	<0.001 −8.3%	<0.001 +8.5%	0.038* −2.3%	0.001* −1.8%
MEP Amplitude – Lower Limb	<0.001 +16.7%	<0.001 +4.1%	<0.001 −7.4%	<0.001 +9.7%	0.046* −2.1%	0.001* −2.1%
CSP – Upper Limb	<0.001 −19.9%	0.017 −14.4%	<0.001 +13.6%	<0.001 −13.9%	1.000 −8.0%	0.0002* +11.1%
CSP – Lower Limb	<0.001 −24.2%	<0.001 −8.4%	<0.001 +15.6%	<0.001 −15.8%	1.000 +1.7%	0.002* +11.6%

*P*-values from Bonferroni-corrected pairwise comparisons. % change = percentage difference between compared timepoints. Bold indicates *p* < 0.05. *Statistically significant difference with negligible absolute magnitude (MEP amplitude <10 μV; MEP latency <0.7 ms); clinical relevance is unlikely. CSP, cortical silent period; MEP, motor evoked potential; MT, motor threshold.

Repeated measures ANOVA demonstrated significant main effects of time point for motor threshold, MEP latency and MEP amplitude for both upper and lower limbs, with large effect sizes ( $\eta_{p}^{2}$ = 0.395–0.854).

Cortical silent period showed a non-significant overall main effect (*p* = 0.132–0.166), despite significant pairwise improvements. Despite the non-significant overall ANOVA effect (*p* = 0.132–0.166), planned pairwise comparisons for CSP were conducted based on *a priori* theoretical predictions of treatment-induced changes in GABAergic inhibition. This approach is justified when specific hypotheses exist regarding directional changes at defined timepoints, independent of the omnibus test (Keppel and Wickens, 2004). The high inter-individual variability in CSP (SD ± 22–44 ms) likely contributed to the non-significant omnibus effect despite robust and consistent pairwise changes. All *p*-values for pairwise comparisons were Bonferroni-corrected for multiple comparisons.

**Figure 1:**
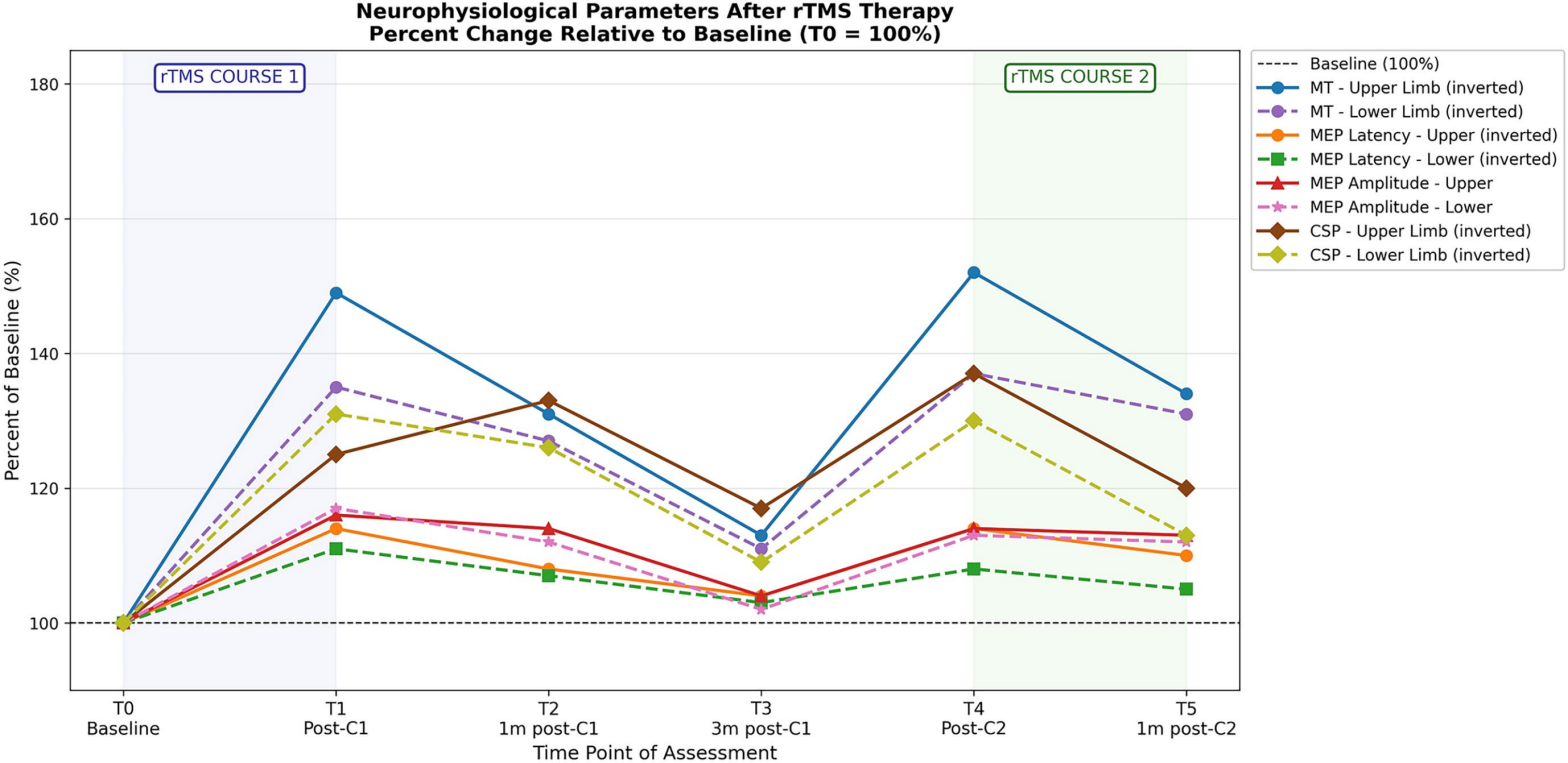
Neurophysiological changes following repeated courses of low-frequency rTMS. The horizontal dashed line at 100% represents baseline at T0. Motor threshold and MEP latency are inverted [% change from baseline * (−1), thus decrease in values is shown as increase in %]. C1 = first rTMS course; C2 = second rTMS course; m = month; MT = motor threshold; MEP = motor evoked potential.

All parameters demonstrated a consistent two-cycle pattern: significant improvement immediately following each course, significant partial decay over the subsequent 3 months, but with sustained improvement compared to baseline in the 3-month follow-up (all *p* ≤ 0.017; Tables 1, 2). The second rTMS course was associated with reproducible effects at 1 month post-course, compared to the first course, with some enhancement in MEP amplitude and CSP. This pattern was identical across upper and lower limb measurements and across both treatment course (Figure 1).

### Functional outcomes

Functional assessments demonstrated significant improvements following rTMS across all five measures, with a consistent two-cycle pattern mirroring the neurophysiological findings (Tables 3, 4). Repeated measures ANOVA revealed large effect sizes for all parameters ( $\eta_{p}^{2}$ = 0.822–0.901), with significant decay during the 3-month follow-up after first course (all *p* ≤ 0.003 for T2 → T3) and maintained improvement over baseline at 3 months (all *p* < 0.001 for T0 → T3). The second rTMS course was associated with reproducible effects at 1 month post course, compared to the first one, but without clinically meaningful enhancement. Complete descriptive statistics are presented in Table 3, and *Post-hoc* pairwise comparisons in Table 4.

**Table 3:** Functional outcomes across six assessment time points.

Parameter	T0	T1	T2	T3	T4	T5	F	*p*	$\eta_{p}^{2}$
Finger Tapping (taps/10s)	27.6 ± 6.3	30.3 ± 6.1	29.4 ± 6.3	28.6 ± 6.2	30.5 ± 6.2	30.1 ± 6.3	79.97	<0.001	0.851
Box and Blocks (blocks)	42.3 ± 7.1	45.9 ± 7.7	44.9 ± 7.5	43.5 ± 7.3	46.3 ± 7.7	45.4 ± 7.5	98.41	<0.001	0.875
TUG (seconds)	9.9 ± 1.8	8.1 ± 1.6	8.2 ± 1.7	8.7 ± 1.7	8.0 ± 1.7	8.1 ± 1.7	64.63	<0.001	0.822
1-Minute Walk (meters)	64.1 ± 16.6	73.9 ± 17.4	73.7 ± 17.6	68.9 ± 16.9	74.1 ± 17.4	73.7 ± 17.6	82.09	<0.001	0.854
Berg Balance (points)	50.5 ± 2.2	53.7 ± 1.8	53.3 ± 2.0	51.8 ± 2.1	53.9 ± 1.7	53.5 ± 1.9	126.85	<0.001	0.901

Values represent mean ± standard deviation. TUG, Timed Up and Go Test (lower values indicate better performance). T0, baseline before first rTMS course; T1, immediately post-first course; T2, 1-month follow-up after first course; T3, 3-months post-first course; T4, immediately post-second course; T5, 1-month follow-up after second course. F-statistic, *p*-value and $\eta_{p}^{2}$ from repeated measures ANOVA. All functional measures showed significant improvements with large effect sizes (*n =* 15). Melbourne Assessment 2 data presented separately in Table 4.

**Table 4:** *Post-hoc* pairwise comparisons of functional outcomes between key timepoints – *p-*value and % change.

Parameter	T0 → T1 (course 1 effect)	T0 → T3 (maintenance)	T2 → T3 (decay)	T3 → T4 (course 2 effect)	T1 vs. T4 (reproducibility)	T2 vs. T5 (enhancement)
Finger Tapping (taps/10s)	<0.001 +9.9%	<0.001 +3.6%	<0.001 −2.7%	<0.001 +6.5%	0.986 +0.4%	0.002* **+2.5%**
Box and Blocks (blocks)	<0.001 +8.7%	<0.001 +3.0%	<0.001 −3.1%	<0.001 +6.3%	0.331 +0.7%	0.411 +1.0%
TUG (seconds)	<0.001 −17.6%	<0.001 −11.8%	0.003 +5.7%	<0.001 −8.4%	0.577 −2.0%	1.000 −1.2%
1-Minute Walk (meters)	<0.001 +15.3%	<0.001 +7.6%	<0.001 −6.4%	<0.001 +7.5%	0.986 +0.4%	— 0.0%
Berg Balance (points)	<0.001 +6.3%	<0.001 +2.5%	<0.001 −2.8%	<0.001 +4.0%	0.986 +0.2%	0.495 +0.4%

*P*-values from Bonferroni-corrected pairwise comparisons. % change = percentage difference between compared timepoints (negative values for TUG indicate improvement—faster time). Bold indicates *p* < 0.05. *Statistically significant difference with negligible absolute magnitude (+0.7 taps/10s); clinical relevance is unlikely. TUG, Timed Up and Go;—= identical values at T2 and T5.

Melbourne Assessment 2 results are reported separately by MACS classification due to ceiling effects in higher-functioning children (Table 5). For MACS I children (*n =* 12), baseline performance was already high across all subscales, with only modest improvements following each course, reflecting limited room for measurable gain in this subgroup. For MACS II children (*n =* 3), more substantial improvements were observed across all four dimensions, particularly in Range and Dexterity subscales. Across both subgroups, subscale scores at T1 were identical to those at T4, consistent with the reproducibility pattern observed for all other outcome measures. Given the very small size of the MACS II subgroup, these findings should be interpreted with caution.

**Table 5:** Melbourne assessment 2 subscale scores by MACS level across six timepoints.

Subscale	MACS	T0	T1	T2	T3	T4	T5
Range (%)	MACS I	89.4 (82.5–93.5)	91.5 (85.5–97.5)	90.5 (84.5–97.5)	90.5 (84.5–97.5)	91.5 (85.5–97.5)	90.5 (84.5–97.5)
	MACS II	69.5 (62.5–84.5)	72.5 (68.5–90.5)	70.0 (65.5–85.5)	70.0 (64.5–85.5)	72.5 (68.5–90.5)	70.0 (65.5–85.5)
Accuracy (%)	MACS I	100 (95–100)	100 (96–100)	100 (95–100)	100 (95–100)	100 (96–100)	100 (95–100)
	MACS II	96 (89–100)	98 (92–100)	96 (90–100)	96 (90–100)	98 (92–100)	96 (90–100)
Dexterity (%)	MACS I	93.5 (87.5–100)	95.5 (89–100)	95.0 (88–100)	94.0 (87–100)	95.5 (89–100)	95.0 (88–100)
	MACS II	69.6 (63.5–83.3)	73.5 (69.5–90.5)	71.0 (66.5–87.5)	70.5 (66.5–86.5)	73.5 (69.5–90.5)	71.0 (66.5–87.5)
Fluency (%)	MACS I	96.4 (88.7–98.0)	98.0 (90.7–99.0)	97.0 (89.5–99.0)	97.0 (89.0–99.0)	98.0 (90.7–99.0)	97.0 (89.5–99.0)
	MACS II	75.5 (65.9–82.0)	78.5 (68.0–84.0)	76.5 (66.5–83.5)	75.5 (66.5–82.5)	78.5 (68.0–84.0)	76.5 (66.5–83.5)

Formal statistical comparisons were not performed due to small subgroup sizes (MACS I: *n =* 12; MACS II: *n =* 3) and non-normal distribution. Scores at T1 = T4 and T2 ≈ T3 ≈ T5 across all subscales and both MACS subgroups, consistent with the reproducibility pattern observed for other outcome measures. Values represent median (IQR).

**Figure 2:**
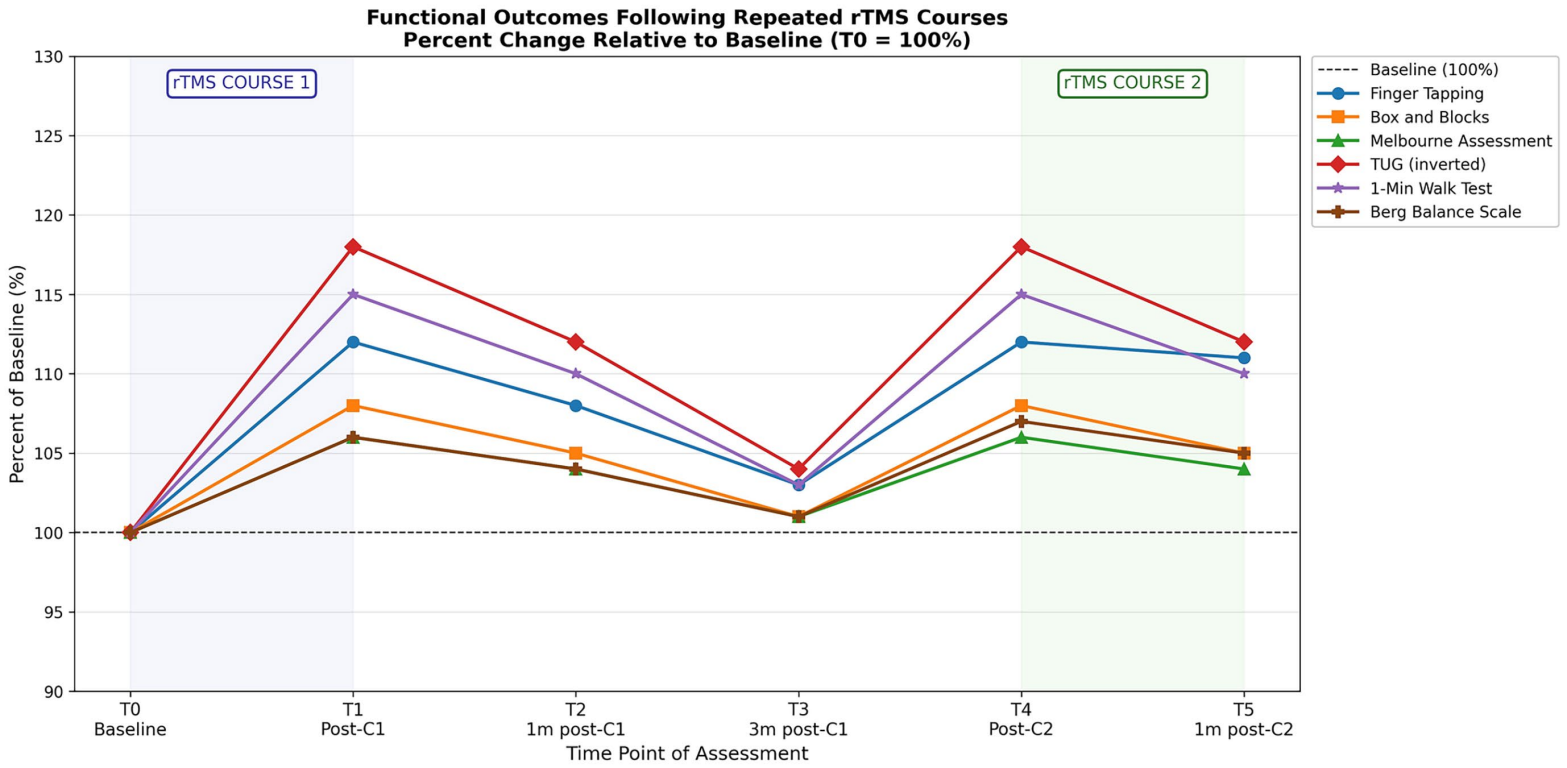
Functional outcomes following repeated rTMS courses.

The temporal dynamics of functional improvements across both rTMS courses are illustrated in Figure 2, demonstrating the reproducible two-cycle pattern with immediate post-treatment gains, partial decay during follow-up, and restoration of effects with the second course.

## Discussion

This prospective exploratory study demonstrates associations between repeated courses of low-frequency rTMS applied to the contralesional motor cortex and sustained modulation of cortical excitability with improvements in motor function in children with hemiplegic cerebral palsy.

A key limitation of this study is the absence of a sham-controlled and blinded design. As a result, the observed improvements cannot definitively be attributed to rTMS. Practice effects from repeated testing, developmental changes over time, regression to the mean, and expectation or placebo effects may all contribute to the observed findings. In addition, functional assessments were performed by unblinded evaluators, introducing further bias. These factors are particularly relevant for measures like the Box and Blocks and Melbourne Assessment, where small differences in scoring may be influenced by observer expectations.

### Neurophysiological effects of contralesional low-frequency rTMS

The substantial decreases in MT observed in both upper and lower limbs following each rTMS course (32.7% reduction for upper limb, 25.8% for lower limb) are consistent with the interhemispheric competition model proposed by Murase et al. (2004) and empirically supported in pediatric populations by Kirton et al. (2008, 2010)). According to this model, reorganization of the contralesional hemisphere in hemiplegic CP results in altered patterns of interhemispheric inhibition, with excessive transcallosal inhibition from the contralesional motor cortex further suppressing motor output to the paretic limbs. By applying inhibitory 1 Hz rTMS to the contralesional motor cortex, we reduced this maladaptive inhibition imposed on the ipsilesional hemisphere. The magnitude of our MT reductions are comparable to those reported by Kirton et al. (2010) in their study of 10 children with perinatal stroke (mean MT decrease of 28%. Our study further extends their findings by demonstrating effect maintenance at multiple follow-up points and reproducibility with a second treatment course. The parallel changes in both upper and lower limb MT suggest diffuse effects beyond the direct stimulation target, consistent with the widespread cortical network modulation hypothesis proposed by Fitzgerald et al. (2006) in their comprehensive review of rTMS effects on motor cortex.

The shortening of MEP latency (11.6% for upper limb, 9.3% for lower limb) and increase in MEP amplitude (16.5% for upper limb, 16.7% for lower limb) provide converging evidence of enhanced corticospinal transmission. Shorter latencies may reflect recruitment of faster-conducting corticospinal pathways, improved synchronization of descending volleys, or enhanced synaptic efficiency at cortical or spinal levels. While activity-dependent myelination has been proposed as a potential mechanism for latency improvements following neuromodulation, this remains speculative and was not directly assessed in our study. The higher post-course MEP amplitudes indicate greater synchronization of corticospinal output, likely reflecting reduced transcallosal inhibition from the contralesional hemisphere. By decreasing contralesional excitability, low-frequency rTMS appears to release the ipsilesional motor cortex from maladaptive suppression, allowing more robust and synchronized corticospinal volleys to the paretic limbs (Di Lazzaro and Rothwell, 2014).

The reduction in CSP duration provides additional evidence of neuroplastic modulation. Planned paired comparisons revealed significant CSP shortening immediately following both treatment courses (19.9–24.2% reduction for first course, *p* < 0.001 for both limbs and both courses). CSP reductions were partially maintained at 3-month follow-up (14.4% for upper limb, *p* = 0.017; 8.4% for lower limb, p < 0.001), with the second course producing virtually identical magnitude of change as the first (T1 vs. T4: *p* = 1.000 for both limbs). The CSP reflects primarily GABA-B mediated intracortical inhibition within the stimulated motor cortex (Ziemann et al., 2015; Stinear et al., 2017). The observed CSP shortening in the ipsilesional hemisphere may indicate rebalancing of local excitatory-inhibitory dynamics following release from excessive transcallosal inhibition, suggesting that contralesional rTMS not only reduces maladaptive interhemispheric inhibition but may also trigger secondary homeostatic plasticity mechanisms within the ipsilesional cortex itself.

Notably, comparison of equivalent post-course timepoints revealed progressive CSP prolongation at T5 compared to T2 for both upper limb (+11.1%, *p* = 0.0002) and also for lower limb (+11.6%, *p* = 0.002), in contrast to the stable pattern observed for all other neurophysiological parameters. This dissociation suggests that GABAergic inhibitory remodeling may accumulate across treatment courses even in the absence of additive changes in corticospinal excitability parameters. However, the high inter-individual variability in CSP measurements (SD ± 22–44 ms) and the non-significant overall ANOVA effect warrant cautious interpretation, and the functional significance of CSP changes in motor improvement remains debated (Chen, 2000; Badawy et al., 2010).

### Functional translation of neurophysiological changes

The improvements observed across multiple functional domains after first rTMS (8.7% more blocks in Box and Blocks, 17.6% faster TUG, 15.3% greater walking distance, 6.3% improved balance) demonstrate that rTMS-induced neurophysiological changes translate into clinically relevant motor gains. Our BBT improvements (mean increase of 3.6 blocks) are comparable to those reported by Gillick et al. (2014) in their randomized trial of primed low-frequency rTMS combined with constraint-induced movement therapy (mean increase of 4.2 blocks) but do not reach the minimal clinically important difference of 7 blocks established for children with hemiplegia (Liang et al., 2021). Nevertheless, the consistent improvements across multiple functional domains and the large statistical effect sizes suggest that rTMS combined with standard physiotherapy produces statistically robust and potentially clinically relevant improvements in manual dexterity, even if individual measures do not cross established MCID thresholds. The functional significance of these sub-MCID improvements, particularly when observed across multiple outcome domains, warrants further investigation in larger controlled trials.

The consistent parallel improvements in both neurophysiological and functional parameters after both rTMS courses suggest a mechanistic link between rTMS-induced modulation of interhemispheric inhibition and improved motor performance. Correlation analyses between individual changes in neurophysiological parameters and functional outcomes did not reach statistical significance for any of the examined pairs (all *p* > 0.05). The strongest trends were observed between reductions in lower limb MT and improvements in TUG (r = +0.42, *p* = 0.119) and Finger Tapping (r = −0.42, p = 0.119). The absence of significant correlations likely reflects the limited statistical power of the present study (*n =* 15) and the relative homogeneity of the cohort (predominantly GMFCS I, MACS I), which restricts the range of individual variability, which is needed to detect inter-individual associations. Larger studies with greater sample sizes and wider functional ranges will be needed to formally characterize the neurophysiological predictors of functional treatment response.

The relatively modest changes in Melbourne Assessment 2, particularly in MACS I children who exhibited ceiling effects, likely reflect the limitations of this measure in detecting change in high-functioning children rather than absence of treatment effect. As noted by Randall et al. (2001) in their validation study, the Melbourne Assessment shows reduced sensitivity at the upper performance range. In contrast, MACS II children demonstrated more substantial improvements, suggesting that rTMS may be particularly beneficial for children with moderate impairment who have greater potential for measurable functional gains.

### Time course of rTMS effects and benefits of repeated courses

#### Duration of treatment effects

A key finding of our study is the maintenance, although partial, of both neurophysiological and functional improvements at 1-month and 3-month follow-up, with some parameters (particularly, MT and MEP amplitude) remaining significantly better than baseline even at T3. This extended duration of effects surpasses the 2–4 week maintenance typically reported in adult stroke studies (18, 25) and aligns with the emerging evidence that developing brain may show durable plastic changes following neuromodulation (Zewdie et al., 2020; Pascual-Leone et al., 2005). Rajapakse and Kirton (2013) proposed that the enhanced neuroplasticity of pediatric brain—characterized by greater synaptic pruning, more extensive dendritic branching, and heightened capacity for structural remodeling, may amplify and prolong rTMS effects compared to adult populations.

#### Is there cumulative effect after two courses of rTMS

To assess whether repeated courses yield cumulative benefits, outcomes at equivalent maintenance timepoints after each course (T2 and T5) were compared and no statistical significance or clinically meaningful difference was found. The notable exception was the CSP, which was significantly longer at T5 compared to T2 for both upper and lower limb. This progressive prolongation of CSP may reflect deepening GABAergic inhibitory remodeling that accumulates across treatment courses, even in the absence of cumulative improvements in corticospinal excitability parameters. This dissociation between inhibitory and excitatory neuroplastic changes suggests that GABAergic mechanisms may be particularly sensitive to repeated rTMS and that cumulative effects, while not universal across all parameters, cannot be excluded.

Thus, the second course of rTMS produced improvements of similar magnitude to the first course across all neurophysiological and functional parameters, demonstrating that repeated rTMS interventions maintain efficacy without diminishing returns. This addresses an important gap in the literature, as most pediatric rTMS studies have examined only single treatment courses (Kirton et al., 2008; Kirton et al., 2016; Gillick et al., 2014). The fact that each course of rTMS produces a similar therapeutic effect, rather than building upon the previous, suggests a ceiling effect for the current stimulation protocol and highlights the potential value of exploring intensified or modified protocols for achieving further functional gains. Further studies with larger sample sizes and a variety of intervals between courses are needed for more solid conclusions on this topic.

The absence of intensive task-specific motor training during the enhanced cortical plasticity window immediately following rTMS represents an important limitation. Studies combining rTMS with constraint-induced movement therapy or other intensive training protocols have demonstrated larger functional gains than rTMS alone (Gillick et al., 2014), suggesting that the neurophysiological changes induced by rTMS create a permissive state for motor learning that may be underutilized without concurrent targeted practice.

### Rationale for maintenance rTMS in pediatric motor rehabilitation

The only partial decay of therapeutic effects by 3 months following an rTMS course provides a physiological rationale for considering scheduled repeated rTMS interventions as a longer-term therapeutic strategy. While maintenance rTMS has been formally investigated in treatment-resistant depression, with protocols involving periodic courses to prevent relapse (Rachid, 2018; Senova et al., 2019), a similar model for pediatric motor rehabilitation has not been described yet.

Our findings, demonstrating that a second rTMS course administered after partial effect decay restored neurophysiological and functional gains to levels at least equivalent to those achieved by the first course, suggest that scheduled repeated courses may represent a viable approach for sustaining motor improvements in children with hemiplegic CP. Such a model, with courses separated by approximately 1–3 months and timed to precede return toward baseline, could provide ongoing support for motor development during critical periods of brain maturation. This hypothesis remains to be tested in prospective studies with longer follow-up and structured re-treatment protocols.

### Mechanisms underlying rTMS effects in developing brain

The mechanisms through which low-frequency rTMS modulates brain function likely involve multiple interacting processes. At synaptic level, 1 Hz stimulation is thought to induce long-term depression (LTD)-like plasticity through mechanisms involving NMDA receptors, calcium-dependent signaling cascades, and metaplastic changes in synaptic efficacy (Fitzgerald et al., 2006; Pascual-Leone et al., 2005). These molecular changes are thought to restructure local cortical circuits progressively in days to weeks following stimulation—a process of synaptic consolidation that may ultimately sustain functional effects well beyond the stimulation period, as evidenced by the maintenance of improvements observed at T2 and T3 in the present study.

At network level, reducing contralesional hyperexcitability rebalances interhemispheric interactions not only through direct transcallosal connections but also via subcortical structures including the thalamus, basal ganglia, and brainstem nuclei that participate in motor control (Kirton and deVeber, 2006; Murase et al., 2004). The broad distribution of functional improvements across both upper and lower limbs, despite the focal stimulation only to the hand-represented cortex, supports involvement of multiple motor networks rather than solely circumscribed cortical changes. This diffuse effect may reflect either direct spread of rTMS effects within motor cortex or indirect modulation through cortico-cortical and cortico-subcortical connections.

The developing brain of children with hemiplegic CP may show enhanced plasticity in response to rTMS compared to adult stroke patients. Staudt (2010) documented extensive cortical reorganization following early brain injury, including ipsilateral corticospinal projections and bilateral motor representations, which may create multiple potential pathways for functional recovery. Eyre et al. (2007) demonstrated that corticospinal tract development continues through adolescence, suggesting an extended therapeutic window during which neuromodulatory interventions might influence pathway maturation and strengthen motor connections.

However, the supposed augmented developmental sensitivity to brain injury also necessitates consideration of potential unintended effects of rTMS on brain maturation. As no adverse effects emerged in our study, as well as in the largest rTMS safety analysis to date with 3.5 million stimulations in children (Zewdie et al., 2020), this method may be considered safe when all precautions and exclusion criteria are applied properly. However, long-term follow-up extending into adolescence and adulthood would provide valuable information about lasting impacts of pediatric rTMS on brain development and function.

### Clinical implications and integration with rehabilitation

Our findings support integration of rTMS into comprehensive rehabilitation programs for children with hemiplegic CP, which is evidence-based for GMFCS and MACS levels I–II by this and other studies (Ziemann et al., 2015; Pascual-Leone et al., 1998; Fitzgerald et al., 2006; Kirton et al., 2008). The optimal inter-course interval remains to be determined: our 3-month interval allowed substantial functional decay before initiating the second course, raising the question of whether shorter intervals (e.g., 6–8 weeks) might better capitalize on neuroplastic mechanisms while minimizing intermediate decline. Integration of rTMS with intensive task-specific training during the period of enhanced cortical plasticity immediately post-stimulation, as demonstrated by Gillick et al. (2014), may further amplify functional benefits.

The comprehensive neurophysiological assessment protocol employed in this study could serve as a model for personalized treatment monitoring. Serial TMS measurements may help to identify children who are responding optimally and others who might benefit from protocol modifications (e.g., different frequencies, intensities, or stimulation targets). Furthermore, baseline neurophysiological characteristics such as presence of MEPs, degree of interhemispheric imbalance, and corticospinal tract integrity as measured by diffusion tensor imaging (Englander et al., 2015; Scheck et al., 2012) might predict treatment response, enabling more targeted patient selection in future applications.

### Recommended assessment battery for clinical practice

Among the neurophysiological parameters assessed in this study, MT and MEP amplitude demonstrated the greatest clinical utility, combining robust effect sizes ( $\eta_{p}^{2}$ = 0.473–0.854), straightforward measurement, and direct reflection of corticospinal excitability changes (Rossini et al., 2015; Groppa et al., 2012). These two parameters are therefore recommended as the core neurophysiological monitoring tools in clinical rTMS practice for children with hemiplegic CP. MEP latency, while statistically significant, showed relatively modest absolute changes (2–3 ms) that may be difficult to interpret clinically outside a specialized neurophysiology setting (Rossini et al., 2015). Cortical silent period, despite showing significant pairwise improvements following each treatment course, demonstrated high inter-individual variability and a non-significant overall ANOVA effect, limiting its utility as a standalone clinical monitoring tool; it remains valuable, however, as a mechanistic research parameter reflecting GABAergic inhibitory remodeling (Ziemann et al., 2015; Chen, 2000).

Among functional assessments, the Timed Up and Go test (Podsiadlo and Richardson, 1991), 1-Minute Walk Test (McDowell et al., 2009), Box and Blocks Test (Mathiowetz et al., 1985), and Berg Balance Scale (Berg et al., 1989) collectively represent the most informative clinical battery, covering the key domains of manual dexterity, functional mobility, walking endurance, and postural stability. These four measures demonstrated the largest effect sizes (Cohen’s d ranging from 2.49 to 4.13) and are widely standardized across pediatric rehabilitation settings. The Melbourne Assessment 2, while valuable in research contexts, showed ceiling effects in children with MACS level I—the majority of our cohort—limiting its sensitivity for detecting treatment-related change in higher-functioning children (Randall et al., 2001). Finger Tapping, although responsive to rTMS effects, provides more limited clinical information beyond the dexterity that is already captured by the Box and Blocks Test (Mathiowetz et al., 1985).

Thus, a pragmatic minimal assessment battery comprising MT, MEP amplitude (Rossini et al., 2015; Groppa et al., 2012), Box and Blocks Test (Mathiowetz et al., 1985), Timed Up and Go (Podsiadlo and Richardson, 1991), and Berg Balance Scale (Berg et al., 1989) would capture the essential neurophysiological and functional dimensions of treatment response within approximately 30–40 min, making it feasible for routine monitoring of repeated rTMS interventions in pediatric hemiplegic CP.

### Comparison with alternative approaches

Our contralesional inhibitory approach represents only one of the several proposed rTMS strategies for hemiplegic CP. Alternative protocols include ipsilesional excitatory stimulation (high-frequency rTMS or theta-burst stimulation) aimed at directly enhancing lesioned hemisphere function, and bilateral stimulation protocols combining contralesional inhibition with ipsilesional facilitation. While direct comparisons are lacking in pediatric populations, adult stroke literature suggests that contralesional inhibitory approaches may be particularly effective when ipsilesional hemispheric damage is severe and/or contralesional hyperexcitability is pronounced (Fregni and Pascual-Leone, 2007; Nowak et al., 2009).

Given that perinatal stroke, a major cause of hemiplegic CP, typically results in substantial structural damage to the affected hemisphere—including cortical volume loss, thinning of the motor cortex, and involvement of the corticospinal tract (Ilves et al., 2014; Stojanovski et al., 2023), the contralesional hemisphere may be expected to assume a compensatory role with altered excitability patterns. In the present study, the consistently elevated MT values in the ipsilesional hemisphere at baseline, together with the robust neurophysiological response to contralesional inhibitory rTMS, provide indirect functional evidence consistent with contralesional hyperexcitability. However, it should be acknowledged that direct neurophysiological assessment of the contralesional hemisphere was not performed in this study, and that bilateral hemispheric involvement is common, particularly in pretermborn children, with one hemisphere typically more affected than the other (Eyre et al., 2007; Staudt, 2010). Future studies incorporating paired-pulse TMS measures of interhemispheric inhibition and neuroimaging would allow more direct characterization of the excitability imbalance prior to treatment.

The choice between contralesional suppression versus ipsilesional facilitation may also depend on the individual patterns of cortical reorganization. Kirton et al. (2010) demonstrated that some children with perinatal stroke develop atypical ipsilateral motor projections from the contralesional hemisphere, for whom suppressive rTMS might be contraindicated. Advanced neuroimaging including diffusion tensor imaging tractography and functional MRI could guide individualized treatment planning by clarifying each patient’s unique pattern of corticospinal organization (Englander et al., 2015; Scheck et al., 2012). Importantly, atypical ipsilateral motor projections can also be identified non-invasively through TMS itself—specifically by mapping ipsilateral MEPs from the contralesional hemisphere and assessing their latency characteristics, which allows differentiation between fastconducting reorganized projections and slow polysynaptic pathways (Eyre et al., 2007).

Transcranial direct current stimulation (tDCS) represents an alternative neuromodulation approach with several practical advantages over rTMS, including lower cost, portability, and most importantly, compatibility with concurrent motor training. Cathodal tDCS applied to the contralesional motor cortex aims to reduce interhemispheric inhibition through a mechanism analogous to low-frequency rTMS. Several pediatric studies have demonstrated feasibility and preliminary efficacy of tDCS in hemiplegic CP, with particular promise when combined with constraint-induced movement therapy (Gillick et al., 2015). However, tDCS effects may be more variable and less focal than rTMS, and optimal stimulation parameters remain under investigation.

The consistent positive response to contralesional rTMS across all participants, with no individual demonstrating functional worsening, is encouraging but does not definitively exclude the presence of atypical ipsilateral motor projections. Given the known heterogeneity of rTMS effects in pediatric CP and the variability in corticospinal reorganization patterns following perinatal injury, the absence of pre-treatment neuroimaging and ipsilateral MEP mapping represents a significant limitation. Future studies should incorporate these assessments to enable individualized treatment planning (Eyre et al., 2007; Staudt, 2010).

### Limitations and future directions

Several limitations of this study should be acknowledged.

The absence of pre-treatment neuroimaging (diffusion tensor imaging tractography) and contralesional hemisphere TMS mapping represents a limitation. While no participant demonstrated worsening in any parameter—suggesting absence of atypical ipsilateral motor organization that would be contraindicated for contralesional inhibition—we cannot formally exclude this possibility. Future studies should incorporate DTI tractography and ipsilateral MEP assessment to screen for atypical corticospinal organization and guide individualized treatment planning.

The protocol did not incorporate intensive task-specific motor training concurrent with rTMS, which may have limited the magnitude and durability of functional improvements. While participants continued routine physiotherapy, the addition of structured, intensive practice during the period of enhanced cortical plasticity following rTMS might amplify and consolidate therapeutic gains.

Important practical barriers limit widespread clinical implementation of rTMS in pediatric rehabilitation. The equipment requires substantial capital investment (€50,000–100,000), specialized training, and dedicated clinical space. The necessity for daily sessions over 2-week periods poses logistical challenges for families, particularly those from rural areas or with limited transportation. Unlike portable neuromodulation alternatives such as tDCS, rTMS equipment cannot be used in home settings or easily integrated with ongoing therapy sessions. The technical requirement for participants to remain still during stimulation precludes concurrent motor training, potentially limiting the synergistic benefits observed when brain stimulation is paired with active practice. These factors may restrict rTMS accessibility primarily to specialized rehabilitation centers in urban settings.

The absence of a control group evokes caution when considering cause-and-effect relationships. Improvements might, at least partially, be due to natural development, learning effects from repeated testing, or placebo responses. However, the magnitude and the immediate timing of effects—maximal improvement immediately post-treatment with gradual decay, argue against mere developmental or learning effects. The consistent pattern of improvement following both rTMS courses further supports the treatment effect hypothesis rather than natural progression.

The relatively small sample size (*n =* 15) and homogeneous population (predominantly GMFCS I, MACS I) limit generalizability to children with more severe impairments or different CP subtypes. Future studies should include larger samples spanning a wider range of functional abilities and CP subtypes to determine optimal patient selection criteria and whether protocol modifications are needed for different subgroups.

The small sample size (*n =* 15) combined with multiple outcome measures and repeated comparisons increases the risk of Type I error. Although corrections were applied, the consistency of statistically significant findings should be interpreted cautiously. Greater emphasis on effect sizes and clinical relevance is warranted, rather than arbitrary *p*-value cut-offs.

Long-term follow-up beyond the 4-month study period is needed to determine whether repeated rTMS courses produce lasting changes in motor development and whether early intervention might alter developmental trajectories. Studies combining rTMS with specific motor training paradigms would help establish optimal integration strategies and potentially reveal synergistic effects. Investigation of rTMS effects on other important outcomes in CP, including spasticity, pain, and activities of daily living, would provide a more comprehensive picture of clinical benefits.

Finally, future research should address the neurobiological mechanisms underlying rTMS effects in the developing brain through integration of advanced neurophysiological techniques (paired-pulse TMS, TMS-EEG), neuroimaging (structural and functional connectivity), and molecular biomarkers (Oberman et al., 2022). Understanding which children are most likely to benefit from rTMS and which brain characteristics predict optimal response will enable more effective clinical translation of this promising intervention.

## Conclusion

In this prospective exploratory study, repeated courses of low-frequency rTMS were associated with within-subject improvements in neurophysiological and functional measures in children with hemiplegic cerebral palsy. These effects appeared to partially decline over time and were restored following a second course of stimulation.

However, given the absence of a sham-controlled and blinded design, these findings should be interpreted as preliminary and hypothesis-generating. The results do not establish efficacy or support routine clinical use of rTMS in this population.

Future randomized, sham-controlled, and blinded trials are required to determine whether rTMS provides benefit beyond natural recovery, practice effects, and other non-specific influences, and to define its role in pediatric neurorehabilitation.

## Data Availability

The original contributions presented in the study are included in the article/supplementary material, further inquiries can be directed to the corresponding author.
